# Structure of Negative Symptoms in Schizophrenia: An Unresolved Issue

**DOI:** 10.3389/fpsyt.2021.785144

**Published:** 2021-12-14

**Authors:** Manuela Russo, Selman Repisti, Biljana Blazhevska Stoilkovska, Stefan Jerotic, Ivan Ristic, Eldina Mesevic Smajic, Fitim Uka, Aliriza Arenliu, Stojan Bajraktarov, Alma Dzubur Kulenovic, Lidija Injac Stevovic, Stefan Priebe, Nikolina Jovanovic

**Affiliations:** ^1^Unit for Social and Community Psychiatry, World Health Organisation Collaborating Centre for Mental Health Services Development, Bart's and The London School of Medicine and Dentistry, Queen Mary University of London, London, United Kingdom; ^2^Clinical Centre, Psychiatric Clinic, University of Montenegro, Podgorica, Montenegro; ^3^University Clinic of Psychiatry, Ss. Cyril and Methodius University in Skopje, Skopje, North Macedonia; ^4^Faculty of Medicine University of Belgrade & Clinic for Psychiatry, University Clinical Centre of Serbia, Belgrade, Serbia; ^5^Department of Psychiatry, Clinical Centre of the University of Sarajevo, Sarajevo, Bosnia and Herzegovina; ^6^Department of Psychology, University of Pristina, Pristina, Kosovo* by United Nations resolution

**Keywords:** negative symptoms, confirmatory factor analysis, CAINS, BNSS, five-factor model, schizophrenia

## Abstract

**Background:** Negative symptoms are core features of schizophrenia and very challenging to be treated. Identification of their structure is crucial to provide a better treatment. Increasing evidence supports the superiority of a five-factor model (alogia, blunted affect, anhedonia, avolition, and asociality as defined by the NMIH-MATRICS Consensus); however, previous data primarily used the Brief Negative Symptoms Scale (BNSS). This study, including a calibration and a cross-validation sample (*n* = 268 and 257, respectively) of participants with schizophrenia, used the Clinical Assessment Interview for Negative Symptoms (CAINS) to explore the latent structure of negative symptoms and to test theoretical and data-driven (from this study) models of negative symptoms.

**Methods:** Exploratory factor analysis (EFA) was carried out to investigate the structure of negative symptoms based on the CAINS. Confirmatory factor analysis (CFA) tested in a cross-validation sample four competing theoretical (one-factor, two-factor, five-factor, and hierarchical factor) models and two EFA-derived models.

**Result:** None of the theoretical models was confirmed with the CFA. A CAINS-rated model from EFA consisting of five factors (expression, motivation for recreational activities, social activities, vocational, and close/intimate relationships) was an excellent fit to the data (comparative fix index = 0.97, Tucker–Lewis index = 0.96, and root mean square error of approximation = 0.07).

**Conclusions:** This study cannot support recent data on the superiority of the five-factor model defined by the NMIH-MATRICS consensus and suggests that an alternative model might be a better fit. More research to confirm the structure of negative symptoms in schizophrenia, and careful methodological consideration, should be warranted before a definitive model can put forward and shape diagnosis and treatment of schizophrenia.

## Introduction

Negative symptoms are predominant features of schizophrenia and deeply affect an individual's level of functioning and quality of life (1). Despite the acknowledgment of the central role of negative symptoms on schizophrenia, there is still a long-standing debate about the nature of their inner composition (2). Over the last decades, the discussion has revolved around two main issues: (1) the ascertainment of negative symptoms as a distinct dimension from other core features of schizophrenia like positive symptoms and disorganization (2–5) and from clinical phenomena (i.e., poor attention) belonging to depression and/or anxiety (6); and (2) the conceptualization of negative symptoms as an elaborate combination of symptoms (multidimensional construct) or as a general (unidimensional) construct (2, 7, 8).

Research focusing on the identification of the number and the structure of factors describing negative symptoms has used exploratory factor analysis (EFA) on symptoms that were mainly assessed with rating scales originally created to evaluate the full spectrum of psychiatric symptoms rather than focusing on negative symptoms. Recent research pinpointed the importance of using second-generation rating scales, such as the Clinical Assessment Interview for Negative Symptoms (CAINS) (9) and the Brief Negative Symptom Scale [BNSS; (10)], which focused exclusively on the assessment of negative symptoms, differentiated between consummatory and anticipatory anhedonia, and excluded other symptoms that were pertinent to other clinical aspects of the illness (i.e., cognition and depression). Moreover, the importance of conducting confirmatory factor analysis (CFA) for drawing conclusion about factorial models was stressed. CFA is a specific statistical approach that aims to test (i.e., accept or reject) theoretical models based on the causal relationship between latent, unobservable constructs (factors) and observed variables [i.e., symptoms (11–13)]. By addressing these two methodological limitations, very recent studies have challenged the statistical and theoretical underpinning of the predominant two-factor model that consists of decreased or lack of motivation and pleasure for relationships and daily activities factor (MAP) and poor expression (behavioral and linguistical deficits) factor (EXP) (9, 14, 15). With the adoption of CFA, recent studies tested four competing theoretical models: a one-factor (unidimensional model), a two-factor (MAP and EXP factors), and a five-factor model based on components (avolition, anhedonia, asociality, blunted affected, and alogia) suggested by the NIMH-MATRICS Consensus (12), and a hierarchical-factor model (MAP and EXP at a higher level and the five factors from the NIMH-MATRICS Consensus). Results consistently found that the two-factor model was inadequate to capture the complexity of negative symptoms while supporting the five-factor model from the NIMH-MATRICS Consensus (11–13, 16–19). Data showed that the five-factor model was superior compared to other theoretical models in chronic, early stage, and prodromal phase of the illness (13, 16, 18). While the one- and two-factor models were poor fit to the data, the hierarchical model was the only one that seemed to be almost equal to the five-factor model (20). Also, the same research group showed that their five-factor model holds across samples of different cultures and languages in a model with 1,691 individuals in the psychosis spectrum (11). The five-factor model was also supported by data obtained by self-report measurement rather than clinician-rated scales (21).

Collectively, available data seem to point out toward the superiority of the five-factor NIMH-MATRICS Consensus model over the others. However, a few points might still warrant attention. Namely, symptoms were almost entirely rated with the BNSS while the CAINS was used only on a subsample of individuals (*n* = 400) (13), and with the exception of a sample from China (11), all data originated from Western and/or high-income countries. These might represent two potential pitfalls: on one side, while the preponderance of findings from mainly one rating scale (i.e., BNSS) might show the potential validity of this scale to theoretically capture and, therefore, measure negative symptoms, it can highlight the need to test the ability of the CAINS, another legitimately valid second-generation scale, to support the model; on the other side, a limited representativeness of countries from previous studies might challenge the extendibility of the model to other, socio-culturally diverse, contexts. There are clinically and research-relevant reasons why the above two issues should be dealt with: a definitive conclusion about the structure of negative symptoms, which could have an impact on future classification and diagnostic instruments, should be representative of the whole clinical population and whether heterogeneity exists in the way symptoms are expressed and rated should be taken into consideration. Consequently, exploring the factorial structure of negative symptoms rated on a scale that is thought to be appropriate to measure a construct is pivotal when that measure is used as an outcome in clinical trials.

In light of the above, the present study has two aims: (1) to explore the factorial structure of CAINS-rated negative symptoms in a calibration sample of schizophrenia (*n* = 268) from the United Kingdom (UK), and (2) to apply a CFA in a sample of schizophrenia or schizoaffective disorder (*n* = 257) from low- and middle-income countries from South Eastern Europe to determine if the five-factor NIMH-MATRICS Consensus model is superior to other theoretical models including one-factor, two-factor, and hierarchical factor models, and to test the data-driven model obtained from EFA in the current sample.

## Methods

### Participants

The study population included 525 participants with schizophrenia or schizoaffective disorder (ICD-10 F20-F25) (22) from two independent samples and studies. The calibration sample, used to explore the factorial structure of CAINS-based negative symptoms, consisted of 268 participants recruited as a part of a multicenter clinical trial across different sites in the UK (23). The cross-validation sample included 257 participants recruited as part of a clinical trial carried out across five South-East European countries (Bosnia and Herzegovina, Kosovo^*^ by UN resolution, Montenegro, North Macedonia, and Serbia) (24).

### Procedures

In both samples, negative symptoms were assessed using the CAINS (9) as part of a more extensive assessment (23, 24). The CAINS is a semi-structured interview rating negative symptoms on 13 items covering the past/next week on a five-point scale from 0 (no impairment) to 4 (severe deficit). The scores are obtained by summing ratings into two main subscales: motivation and pleasure (MAP; items 1–9) and expression (EXP; items 10–13). The CAINS was administered by trained researchers, and an excellent inter-rater reliability was obtained among raters in the cross-validation sample (intraclass correlation coefficient of 0.98). Inter-rater reliability was not available in the calibration sample.

Data were collected during interviews between December 2011–June 2013 (calibration sample) and January 2019–April 2019 (cross-validation sample). Both studies were approved by relevant ethics committees and participants provided written informed consent (23, 24).

### Data Analysis

Descriptive statistics were used to describe demographic and clinical characteristics of participants. The data analysis consisted of two stages: exploring factorial models based on CAINS-rating negative symptoms in the calibration sample using EFA (stage 1); adopting CFA in the cross-validation sample to determine if the five-factor NIMH-MATRICS Consensus model was the best among competing theoretical factorial models. Finally, as two potentially valid factor models emerged from the EFA (see Results session), they were tested in the CFA along with theoretical models (stage 2).

In stage 1, EFA using principal axis factoring, with promax (oblique) rotation, was carried out in the calibration sample using the 13 items of the CAINS. The Kaiser-Meyer-Olkin (KMO) and Bartlett's test of sphericity were used to measure sampling adequacy with acceptable values being equal to or higher than 0.6 (25). To examine whether data could reflect factors' composition of the major theoretical models, a series of EFA were run and set to extract a fixed number of factors that were, in turn, one (unidimensional model), two (MAP and EXP model), and five (NIMH-MATRICS Consensus model). Finally, an eigenvalue of at least 1 was used as extraction method to explore the natural aggregation of items.

In stage 2, CFA was applied to test goodness of fit of theoretical models and the models obtained from EFA at stage 1. Before running the CFA, the cross-validation sample was checked for missing data and multivariate normal distribution (as this latter is an assumption for CFA). We adopted a conservative approach that consisted in the deletion of cases that had missing data for any CAINS item; outliers identified with a Mahalanobis distance value significant at *p* < 0.05 (indicator of outliers) were also excluded. This approach resulted in the current sample size of 257. Next, CFA was carried out with Maximum Likelihood estimation method, and the following indices were used to determine goodness of fit: root mean square error of approximation (RMSEA; acceptable value <0.08), comparative fit index (CFI), and the Tucker–Lewis index (TLI) (acceptable values ≥0.95) (26); standardized root mean square residual (SRMR; acceptable value ≤0.08); adjusted goodness-of-fit index (AGFI; acceptable value <0.95) (26); and relative χ^2^ (consisting of the ratio between χ^2^ and the degree of freedom), with recommended value <3 (26). Finally, to determine the best among the competing models, the Akaike information criterion (AIC) and the Bayesian information criteria (BIC) were used with lower values indicating better fit. All factorial models will be graphically depicted with values for standardized regression weights [identifiable as arrows from observable (squared objects) variables, CAINS' items, to latent variables (oval objects) and factors]. The CFA analysis was then repeated excluding the sample participants with schizoaffective disorder (*n* = 31) in order to reduce potential symptoms' heterogeneity. All analyses were carried out with SPSS Statistics and AMOS (both versions 27).

## Results

Socio-demographic and clinical characteristics of the two samples are reported in Table 1. The two samples are different across all aspects but number of hospitalizations; participants in the calibration sample were younger, with both higher level of education and rates of unemployment. Also, in the calibration sample, the proportion of men was lower, diagnosis was exclusively of schizophrenia, and negative symptoms were more severe than in the cross-validation sample.

**Table 1:** Description and comparison between the calibration and cross-validation sample.

	**Calibration sample (*n* = 268)**	**Cross-validation sample (*n* = 257)**	**Statistics**
Age, mean (SD)	42.2 (10.6)	44.0 (10.2)	*t*(537) = 2.10; *p* = 0.036
Female, *N* (%)	68 (25.4)	101 (39.3)	χ^2^(1) = 11.7; *p* < 0.001
Diagnosis, *N* (%)			χ^2^(1) = 34.4; *p* < 0.001
Schizophrenia	268 (100.0)	226 (87.9)	
Schizoaffective	–	31 (12.1)	
Number of psychiatric hospitalizations, mean (SD)	4.0 (4.0)	3.8 (3.7)	*t*(453) = −0.4; *p* = 0.713
CAINS, mean (SD)			
MAP	21.7 (5.6)	16.5 (8.4)	*t*(523) = −8.5; *p* < 0.001
EXP	7.8 (3.7)	4.7 (3.9)	*t*(523) = −9.4; *p* < 0.001
Level of education, *N* (%)			χ^2^(2) = 71.4; *p* < 0.001
Elementary and less	3 (1.2)	57 (22.2)	
Secondary School	165 (63.7)	163 (63.4)	
Graduate and postgraduate	91 (35.1)	37 (14.4)	
Employment, *N* (%)			χ^2^(3) = 83.8; *p* < 0.001
Paid/sheltered employment	2 (0.7)	29 (11.3)	
Training/studying	2 (0.7)	6 (2.3)	
Unemployed	257 (96.3)	167 (65.0)	
Retired/other	6 (2.2)	55 (21.4)	

*Stage 1*: Four factorial models, consisting of one-, two-, four-, and five-factor models, emerged from the EFA (KMO and Bartlett's test = 0.83, *p* < 0.001) and are reported in Table 2. The magnitude of the loadings varied across models; they were consistently high in the five-factor model, which, differently from the other models, did not emerge with any loadings lower than the absolute reference threshold of 0.40 (25). Conversely, the one- and the two-factor models had 7 and 4 out of 13 items, respectively, with a factor loading <0.4, and only 2 out of 13 items in the four-factor model. From inspection of items segregation, it was noted that while factors of the two-factor model reflected the theoretical model of MAP and EXP factors, factors of the five-factor model did not show same items' segregation than the five-factor NIMH-MATRICS Consensus model. In summary, from the EFA, factors' composition of the one- and two-factor models overlapped with relative theoretical models, while the other two models obtained from EFA (with four and five factors) did not reflect any theoretical models.

**Table 2:** One-, two-, and five-factor model derived from exploratory factor analysis.

**First-order factor analysis**							
One-factor model^*^		Two-factor model^**^		Four-factor model^***^		Five-factor model^**^	
**Items**	**Factor loading**	**Items**	**Factor loading**	**Items**	**Factor loading**	**Items**	**Factor loading**
**Factor 1**		**Factor 1**		**Factor 1**		**Factor 1**	
CAINS_8	0.640	CAINS_11	0.903	CAINS_11	0.898	CAINS_11	0.924
CAINS_4	0.621	CAINS_12	0.864	CAINS_12	0.855	CAINS_12	0.854
CAINS_9	0.616	CAINS_10	0.766	CAINS_10	0.769	CAINS_10	0.763
CAINS_3	0.616	CAINS_13	0.626	CAINS_13	0.633	CAINS_13	0.611
CAINS_7	0.541	**Factor 2**		**Factor 2**		**Factor 2**	
CAINS_2	0.475	CAINS_8	0.708	CAINS_8	0.947	CAINS_9	0.955
CAINS_11	0.326	CAINS_4	0.668	CAINS_9	0.687	CAINS_8	0.682
CAINS_1	0.323	CAINS_9	0.647	CAINS_7	0.638	CAINS_7	0.638
CAINS_13	0.304	CAINS_3	0.642	**Factor 3**		**Factor 3**	
CAINS_12	0.297	CAINS_7	0.624	CAINS_3	0.960	CAINS_4	0.878
CAINS_5	0.293	CAINS_2	0.389	CAINS_4	0.715	CAINS_3	0.769
CAINS_10	0.262	CAINS_5	0.370	CAINS_2	0.345	**Factor 4**	
CAINS_6	0.233	CAINS_6	0.312	CAINS_1	0.304	CAINS_6	0.827
		CAINS_1	0.257	**Factor 4**		CAINS_5	0.734
				CAINS_5	0.888	**Factor 5**	
				CAINS_6	0.666	CAINS_1	0.582
						CAINS_2	0.447

*CAINS_1 (Motivation for close family/spouse/partner relationships), CAINS_2 (Motivation for close friendships and romantic relationships), CAINS_3 (Pleasure social activities, past-week pleasure), CAINS_4 (Social activities, expected pleasure), CAINS_5 (Vocational, motivation), CAINS_6 (Vocational, expected pleasure), CAINS_7 (Motivation for recreational activities), CAINS_8 (Recreational activities, past-week pleasure), CAINS_9 (Recreational activities, expected pleasure), CAINS_10 (Facial expression), CAINS_11 (Vocal expression), CAINS_12 (Expressive gestures), CAINS_13 (Quantity of speech)*.

* *Principal axis factoring, with no rotation, fixed number of factors as extraction method*.

** *Principal axis factoring, with Promax rotation, fixed number of factors as extraction method*.

*** *Principal axis factoring, with Promax rotation, with eigenvalue >1 as extraction method*.

*Stage 2:* CFA used the cross-validation sample to test goodness of fit on the following four theoretical models: a one-, two-, and five-factor NIMH-MATRICS Consensus model, and a hierarchical model (i.e., two high-order factors, MAP and EXP, and the five factors from the NIMH-MATRICS Consensus) (Supplementary Material 1). Next, as results from our EFA in stage 1 showed a four-factor and a five-factor model (this latter was different from the five-factor NIMH-MATRICS Consensus), these two exploratory models were tested in the CFA.

Based on the examination of fit indices, all theoretical models did not meet acceptance criteria for goodness of fit (Table 3), thus failing to represent good fit to the data. Among them, the unidimensional model was the poorest one, while the five-factor model showed the best indices (but not good enough to reach acceptance's threshold). Conversely, the five-factor model obtained from the EFA from this study emerged with excellent fit to the data (Table 3). All standardized regression weights were in the highest range (above 0.8) with the weakest being 0.67 between F5 and item 1 of the CAINS (Supplementary Material 1). The four-factor model presented with good indices, but in the borderline level for acceptability and not as good as those in the five-factor model. Results were confirmed when CFA was carried out, excluding participants with a diagnosis of schizoaffective disorder (Supplementary Material 2).

**Table 3:** Goodness-of-fit indices of factorial models based on theories and on exploratory factor analysis (EFA) of negative symptoms.

**Factorial model**	**CMIN/df**	**CFI**	**TLI**	**AIC**	**BIC**	**RMSEA**	**SRMR**	**AGFI**
**Theoretical**								
One-factor	18.557	0.509	0.420	1,274.759	1,277.652	0.262	0.1032	0.374
Two-factor	10.606	0.740	0.683	732.790	735.914	0.194	0.1020	0.586
Five-factor (NIMH-MATRICS consensus)	10.442	0.768	0.688	673.637	677.571	0.192	0.0945	0.601
Hierarchical model (two high-order factors—MAP and EXP—with	9.648	0.740	0.714	727.035	729.464	0.184	—	0.631
five-factor model from NIHM Consensus)								
**Based on EFA**								
Four-factor	3.316	0.942	0.924	259.622	263.325	0.095	0.0670	0.830
Five-factor	2.312	0.969	0.957	199.148	203.313	0.072	0.0366	0.888

## Discussion

The main finding from this study is that the five-factor model of negative symptoms based on components suggested by the NIMH-MATRICS Consensus (i.e., alogia, blunted affect, anhedonia, asociality, and avolition) could not be confirmed as the best model to conceptualize the structure of negative symptoms in schizophrenia. Likewise, the other theoretical models—one-factor, two-factor (MAP and EXP), and hierarchical model of negative symptoms—failed to be good fit to the data. Conversely, our EFA suggested that the factorial structure of CAINS-rated negative symptoms can be defined by an alternative five-factor model (all factors were robust as they were in the high range and none below the threshold of 0.4; Table 2). This model was then confirmed as excellent fit in the CFA showing that all standardized regression weights were high (equal to or above 0.7), thus suggesting that each latent factor accounted for a very good amount of variance of the items loading on it. Importantly, the fact that the model was held in an independent (and different in terms of socio-demographic characteristics and severity of negative symptoms) sample of schizophrenia is a strong indicator that it is a good model of negative symptoms. Specifically, the following factors emerged, which, based on the items' composition, could be labeled as “expression” (Factor 1), “motivation for recreational activities” (Factor 2), “motivation for social activities” (Factor 3), “motivation for vocational activities” (Factor 4), and “motivation for closer relationships” (Factor 5).

To the best of our knowledge, the current study is the first among recent CFA studies on the latent structure of negative symptoms that does not corroborate the five-factor NIMH-MATRICS Consensus model (11, 13, 16, 18). Two potential reasons can be put forward to explain this discrepancy: the main reason is methodological, related to the different rating scale used, while the second is cultural.

In our study, the CAINS was used to measure negative symptoms, while previous data, with the only exception of a subsample in Strauss et al. (13), exclusively employed the BNSS. Although the original intent behind the development of both CAINS and BNSS was to create next-generation scales able to concisely capture negative symptoms to be used in multisite clinical trials and across culture while covering the five components suggested by the NIMH-MATRICS consensus (9, 27), the two scales have some differences (28) and therefore might measure distinct facets of negative symptoms. Alogia, for example, is covered by one item in the CAINS and two items (quantity of speech and spontaneous elaboration) in the BNSS. The CAINS has an overall stronger emphasis on asociality, which is measured separately across different types of relationship, while the BNSS rates them as a whole. Avolition in the CAINS is measured in relation to working/studying and recreational activities, while the BNSS has separate items for inner experience and behavior (6, 28). However, the wider difference, as previously demonstrated by low correlations between items of interest, is observed on anhedonia, which is based on frequency (detailed account, not prompted by the rater) in the CAINS, and on a broader recall across different domains considering both frequency and intensity in the BNSS (28). Also, the layout of manual/scoresheet of the CAINS (items grouped together in relation to separate social domains in the rating evaluation form) might have contributed to the halo effect on rating and therefore on factors' composition in our study. Finally, potential differences in the estimation method used in conducting the CFA (Maximum Likelihood vs. Weighted Least Square with Mean and Variance adjusted) might account for discrepancy between our results and previous data.

In light of these considerations, it is plausible to think that differences between BNSS and CAINS can be accountable for the discrepancy between our findings and other recent CFA data; nevertheless, findings might not be mutually exclusive and hold different, but equally valid, perspectives about the structure of negative symptoms. Possibly, the structure of the BNSS aligns better than the CAINS with the five components of the NIMH-MATRICS Consensus. Indeed, while at least two items of the BNSS cover each aspect of negative symptoms as defined by the NIMH-MATRICS, only one item in the CAINS covers alogia, and two items cover asociality and amotivation. Given the complexity of this psychopathological phenomena, a future wiser approach should consider combining and analyzing data from different scales measuring negative symptoms. In this way, the latent structure of negative symptoms would be based on a more comprehensive assessment that would take into account all potential facets of their components and, importantly, will be independent from the rating scale. It is also important to highlight that with the exception of blunted affect and alogia, which across data emerged as robust factor/s thus corroborating the traditional view of primary negative symptoms (29), the other components are somewhat inconsistent. Anhedonia and asociality might not be primary negative symptoms, but rather secondary symptoms associated to comorbid depression and psychosis (30). Evidence suggests that anhedonia is characteristic of schizophrenia only in relation to future pleasurable stimuli (anticipatory anhedonia), but not for ongoing pleasant stimuli [consummatory anhedonia; (31, 32)], and that asociality can be a consequence, not only of psychotic symptoms, but also of abnormal social cognition [e.g., deficits in processing and responding to social stimuli; (33, 34)]. Lastly, avolition is a broad and articulate construct whose pathophysiology embeds different high-order cognitive abilities like goal-directed actions, reinforcement learning, reward prediction, and decision-making tapping on different neural networks (35–38).

Cultural differences might also account for divergence of our results from previous data. Although there is evidence that the five-factor NIMH-MATRICS Consensus model holds across different cultures and languages (11), the current study is the first of its kind utilizing a sample from low- and middle-income countries in South East Europe. It could be possible that this sample presents some cultural specificities in the way negative symptoms are interpreted and expressed, which do not fit the traditional view, expression, and report of negative symptoms. Contextual factors like socio-economic status, employability, and social and community resources might influence the way negative symptoms, especially in relation to avolition and amotivation, are reported and rated. However, this remains a mere speculation as there are not enough data to fully support this claim. Indeed, there is an urgent need to conduct research in Central and South East Europe, which is considered a “blind spot” in global mental health map (39).

Our study has several strengths. This study is the second CFA study, after Strauss et al. (13), that looks at the latent structure of negative symptoms with the use of the CAINS. To confirm competing models in the CFA, we used an independent, cross-validation sample from a diverse socio-cultural background that allowed to demonstrate that our model is valid and independent from potential specificities of the calibration sample. The use of a UK/English-speaking calibration sample allowed us to be confident about avoiding any potential bias that might arise because of the use of a non-English, translated version of the CAINS. Finally, this study is the first to test the structure of negative symptoms using the largest sample of individuals with schizophrenia in South Eastern Europe and, consequently, contributes to the global effort to improve research (and mental healthcare) in a developing region.

However, we also acknowledge some limitations. First, only chronic individuals with schizophrenia from outpatient services were included in this study; therefore, caution should be used when extending results from both the EFA and CFA to other (earlier) stages of the illness. Second, the inter-rater reliability among CAINS raters was only available for the cross-validation sample. Third, possibly a more powerful statistical software for CFA might have allowed for different estimation methods known to be superior in handling ordinal variables like the ones of interest.

In conclusion, this study did not support any definitive claim about which theoretical model best describes the factorial structure of negative symptoms in schizophrenia. Neither the five-factor model from the NIMH-MATRICS Consensus nor the other theoretical factor models of negative symptoms in schizophrenia could be confirmed from this study; however, our results suggested that an alternative five-factor model might define the structure of CAINS-rated negative symptoms. Methodological reasons related to the use CAINS and BNSS might be accountable for this inconclusiveness. Further research addressing potential methodological issues should be warranted before moving toward an in-depth revision of the current conceptualization of negative symptoms in schizophrenia, its diagnosis, and treatment.

## Data Availability Statement

The raw data supporting the conclusions of this article will be made available by the authors, without undue reservation.

## Ethics Statement

The studies involving human participants were reviewed and approved by Bosnia and Herzegovina (Klinicki Centar Univerziteta u Sarajevu—Eticki Komitet 03-02-216, Eticki komitet JU Psihijatriska bolnica Kantona Sarajevo and JU Zavod za bolesti ovisnosti Kantona Sarajevo 02.8–408/19), Kosovo^*^ (Hospital and University Clinical Service of Kosovo—Ethics Committee 2019-85), Montenegro (Javna Zdravstvena Ustanova Klinicki Centar Crne Gore—Eticki komitet 03/01–29304/1, ZU Specijalna Bolnica za Psihijatriju Dobrota Kotor—Eticki komitet, Eticki Komitet JZU Dom Zdravlja Dr. Nika Labovic Berane 01-47), Republic of North Macedonia (Eticka Komisija za istrazuvanje na luge, Medicinski Fakultet pri UKIM vo Skopje 03-24219), and Serbia (Eticka komisija Medicinskog fakulteta u Beogradu 2650/XII-20 and Eticka komisija Specijalne bolnice Dr. Slavoljub Bakalovic Vrsac 01-36/1) and the United Kingdom (Queen Mary University of London QMREC2204a, 16 October 2018). The patients/participants provided their written informed consent to participate in this study.

## Author Contributions

MR developed the research idea, conducted statistical analysis, interpreted results, and wrote the first and final full draft. SR, BB, IR, and SJ collected data and contributed to interpretation of results. SP and NJ contributed to critical evaluation of the research idea. EM, FU, AA, SB, AD, and LI critically revised the final full draft. All authors read and approved the final version of the manuscript.

## Funding

This study has received funding from the European Union's Horizon 2020 research and innovation programme under grant agreement no. 779334.

## Conflict of Interest

The authors declare that the research was conducted in the absence of any commercial or financial relationships that could be construed as a potential conflict of interest.

## Publisher's Note

All claims expressed in this article are solely those of the authors and do not necessarily represent those of their affiliated organizations, or those of the publisher, the editors and the reviewers. Any product that may be evaluated in this article, or claim that may be made by its manufacturer, is not guaranteed or endorsed by the publisher.
