# Corrigendum: Structure of Negative Symptoms in Schizophrenia: An Unresolved Issue

**DOI:** 10.3389/fpsyt.2022.885883

**Published:** 2022-03-28

**Authors:** Manuela Russo, Selman Repisti, Biljana Blazhevska Stoilkovska, Stefan Jerotic, Ivan Ristic, Eldina Mesevic Smajic, Fitim Uka, Aliriza Arenliu, Stojan Bajraktarov, Alma Dzubur Kulenovic, Lidija Injac Stevovic, Stefan Priebe, Nikolina Jovanovic

**Affiliations:** ^1^Unit for Social and Community Psychiatry, World Health Organisation Collaborating Centre for Mental Health Services Development, Bart's and The London School of Medicine and Dentistry, Queen Mary University of London, London, United Kingdom; ^2^Clinical Centre, Psychiatric Clinic, University of Montenegro, Podgorica, Montenegro; ^3^University Clinic of Psychiatry, Ss. Cyril and Methodius University in Skopje, Skopje, North Macedonia; ^4^Faculty of Medicine University of Belgrade & Clinic for Psychiatry, University Clinical Centre of Serbia, Belgrade, Serbia; ^5^Department of Psychiatry, Clinical Centre of the University of Sarajevo, Sarajevo, Bosnia and Herzegovina; ^6^Department of Psychology, University of Pristina, Pristina, Kosovo* by United Nations resolution

**Keywords:** negative symptoms, confirmatory factor analysis, CAINS, BNSS, five-factor model, schizophrenia

In the published article, there was an error in affiliation number 6. Instead of “Department of Psychology, University of Pristina, Pristina, Albania,” it should be “Department of Psychology, University of Pristina, Pristina, Kosovo^*^ by United Nations resolution.”

The authors apologize for this error and state that this does not change the scientific conclusions of the article in any way. The original article has been updated.

## Publisher's Note

All claims expressed in this article are solely those of the authors and do not necessarily represent those of their affiliated organizations, or those of the publisher, the editors and the reviewers. Any product that may be evaluated in this article, or claim that may be made by its manufacturer, is not guaranteed or endorsed by the publisher.

