# Supplementary material for: Structure of Negative Symptoms in Schizophrenia: An Unresolved Issue
Source: Front Psychiatry. 2021 Dec 14;12:785144. doi: 10.3389/fpsyt.2021.785144 (PMC8712471; doi:10.3389/fpsyt.2021.785144)
Supplement: Supplementary file 1 [file Data_Sheet_1.docx]

| ***Supplemental material 1*** *Graphical depiction with regressions coefficients of theoretical models of one-, two-, five-, and hierarchical-factor models (a, b, c, d), and of data-driven (obtained from exploratory factor analysis in the current sample) four-, and five-factor models (e, f)* | | |
| --- | --- | --- |
| 1. *theoretical one-factor model* | 1. *theoretical two-factor model* | 1. *theoretical five-factor model* |
| *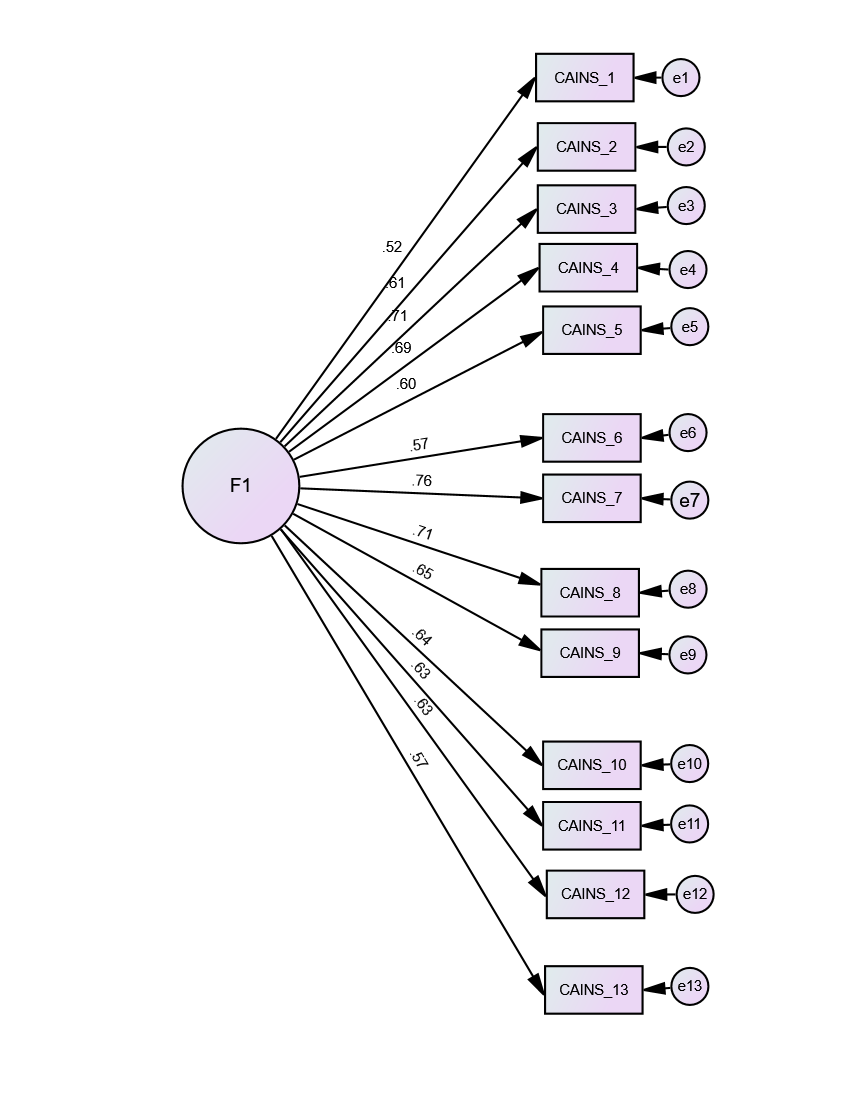* | *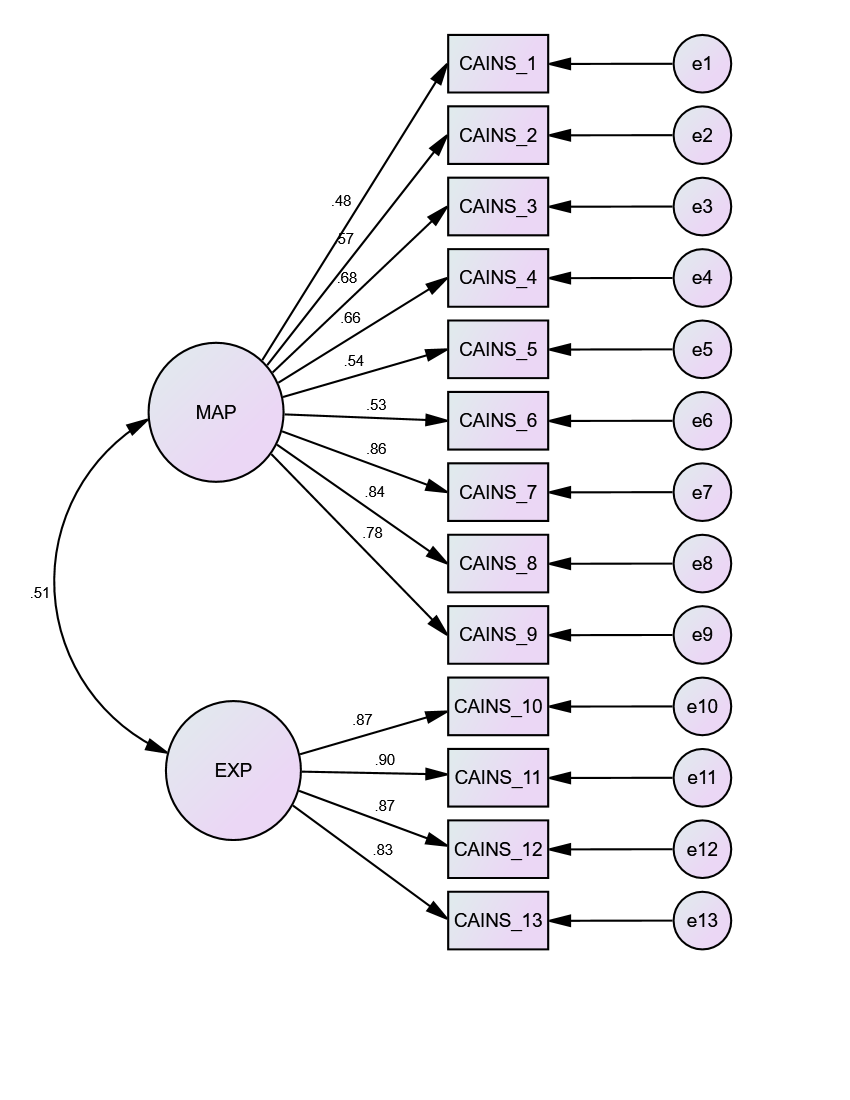* | *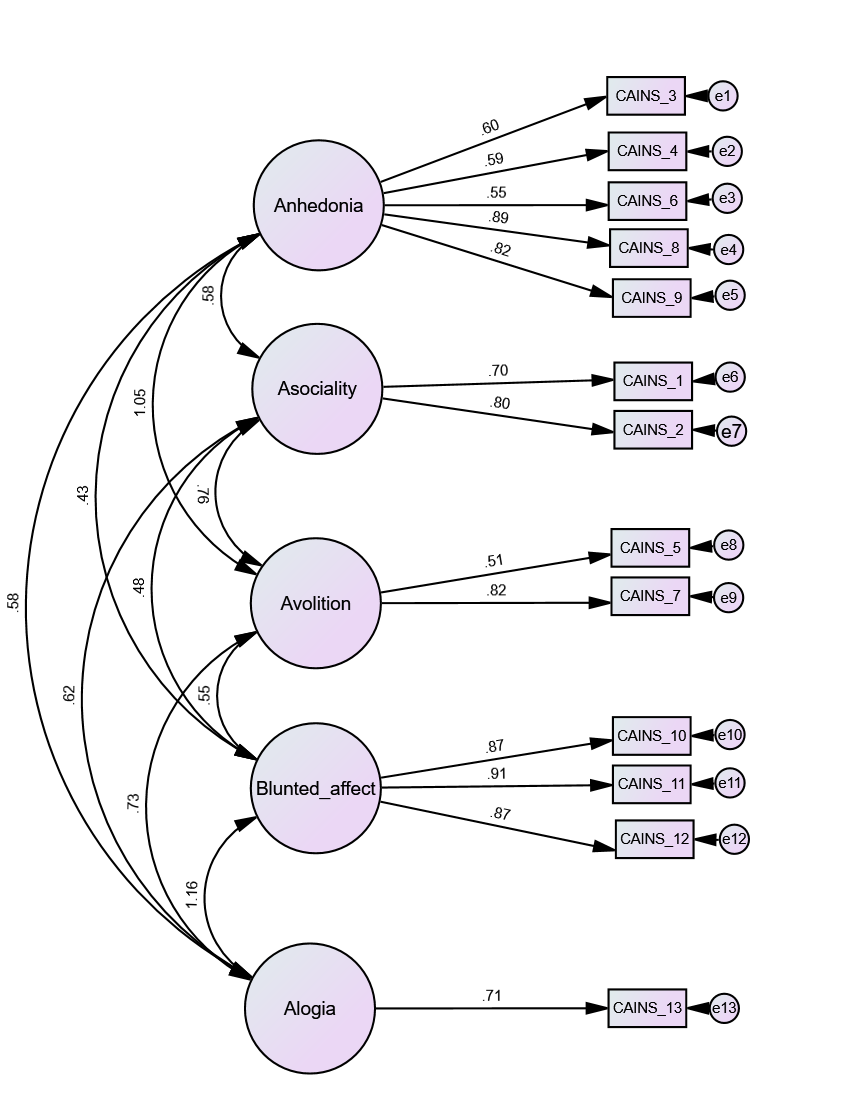* |
|  |  |  |
| *d) theoretical hierarchical-factor models* | *e) data-driven four-factor model* | *f) data-driven five-factor model* |
| *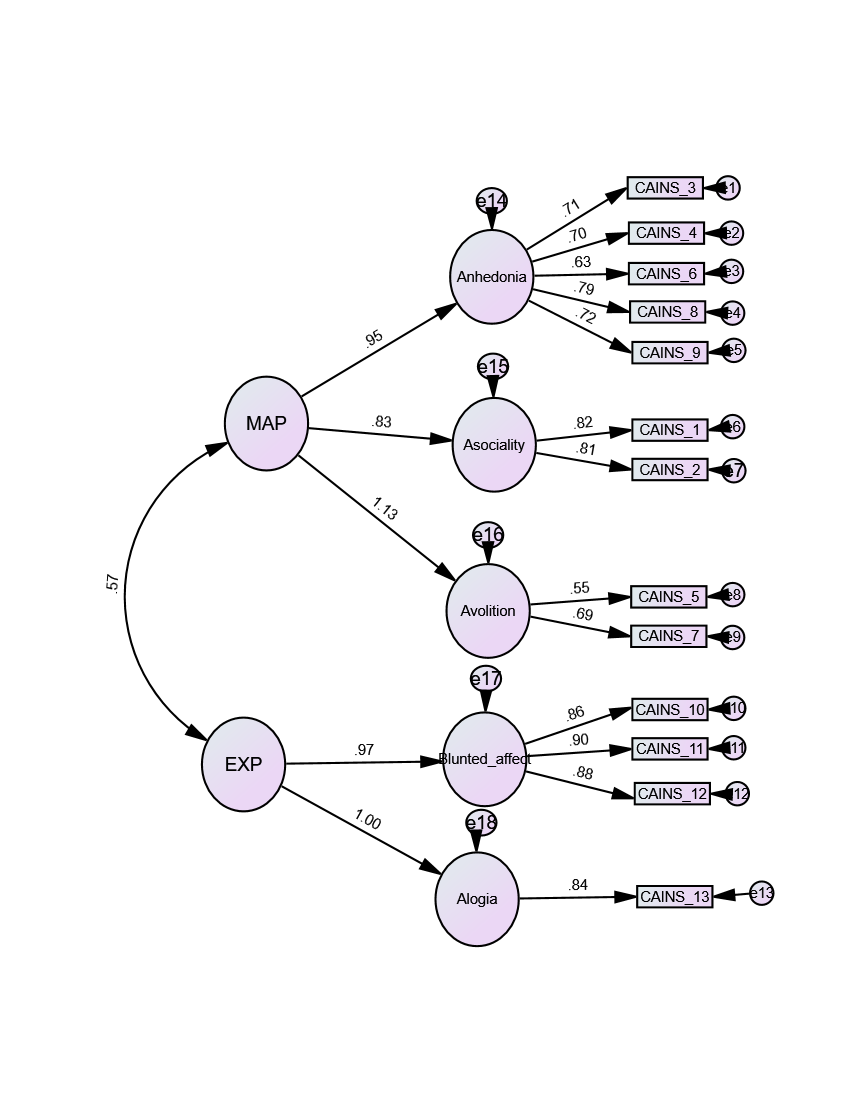* | *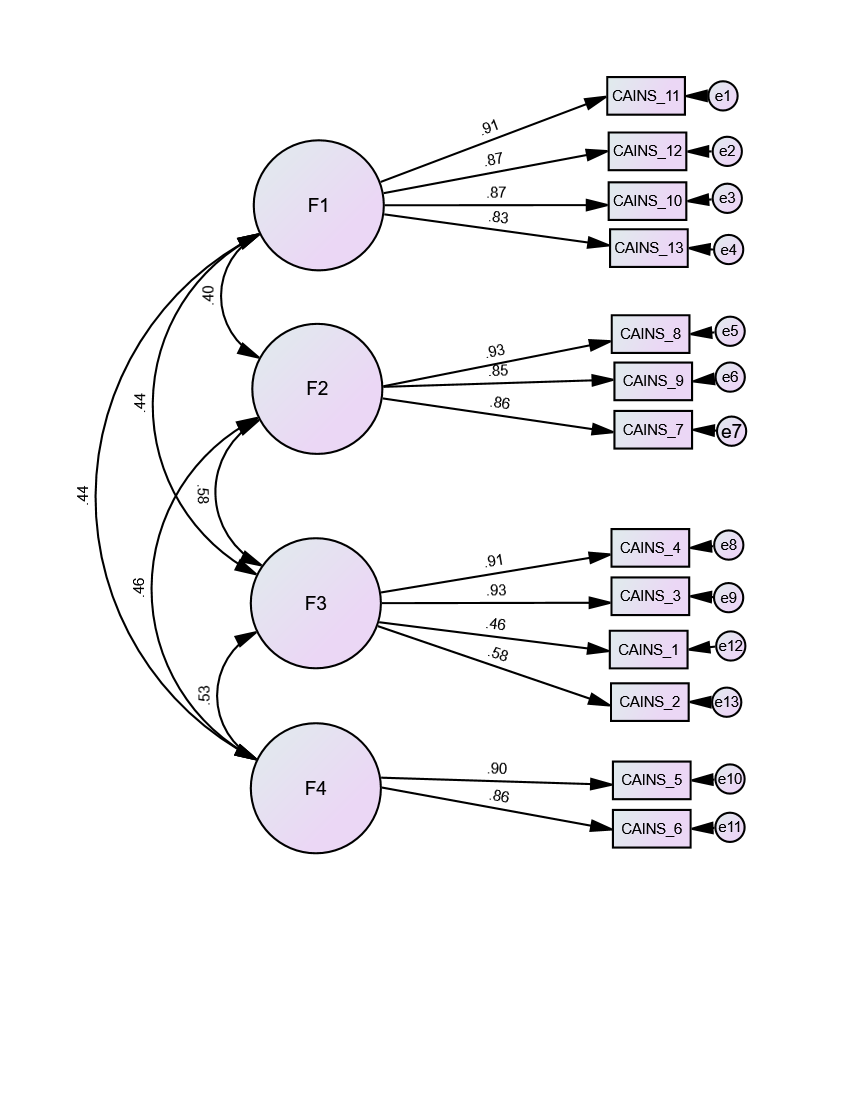* | *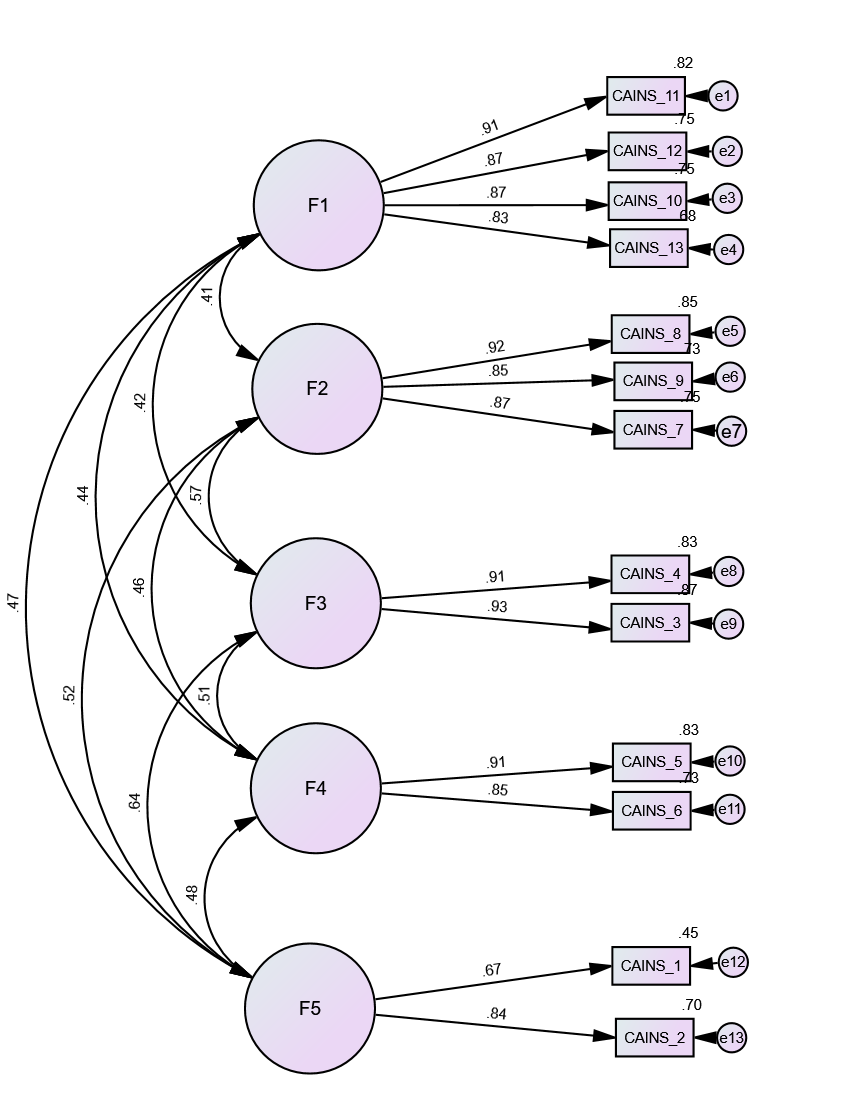* |

**Supplemental material 2**  Goodness-of-fit indices of factorial models based on theoretical and on exploratory factor analysis (EFA) of negative symptoms using only subjects with diagnosis of schizophrenia (excluding schizoaffective diagnosis)

| Factorial model | CMIN/df | CFI | TLI | AIC | BIC | RMSEA | SRMR | AGFI |
| --- | --- | --- | --- | --- | --- | --- | --- | --- |
| *Theoretical* |  |  |  |  |  |  |  |  |
| One-factor | 16.103 | 0.514 | 0.425 | 1112.781 | 1198.294 | 0.295 | 0.1391 | 0.374 |
| Two-factor | 9.352 | 0.739 | 0.682 | 652.538 | 744.892 | 0.193 | 0.1035 | 0.585 |
| Five-factor (NIMH- MATRICS consensus) | 8.346 | 0.756 | 0.720 | 613.533 | 692.205 | 0.181 | 0.0965 | 0.623 |
| Hierarchical model (two high order factors -MAP and EXP - with five-factor model from NIHM Consensus) | 12.540 | 0.561 | 0.561 | 1004.089 | 1005.814 | 0.226 | 0.1471 | 0.534 |
| *Based on EFA* |  |  |  |  |  |  |  |  |
| Four-factor | 2.904 | 0.945 | 0.928 | 235.339 | 344.796 | 0.092 | 0.0667 | 0.832 |
| Five-factor | 2.023 | 0.973 | 0.961 | 183.291 | 306.430 | 0.067 | 0.0382 | 0.887 |
